# Vemurafenib as bridging therapy of hairy cell leukemia in a Jehovah’s Witness patient

**DOI:** 10.1007/s00277-021-04403-4

**Published:** 2021-01-12

**Authors:** Sebastian Schlaweck, Peter Brossart, Annkristin Heine

**Affiliations:** Medical Clinic III for Oncology, Hematology, Immune-Oncology and Rheumatology, University Hospital Bonn, Venusberg Campus 1, 53127 Bonn, Germany

Dear Editor,

A 42-year-old woman with no relevant co-morbidities was admitted to our hospital due to symptomatic anemia. Initial blood counts revealed regular white blood cells (5.49 G/l), but not measurable absolute neutrophil count (ANC), severe symptomatic anemia (6.6 g/dl), and thrombocytopenia (51G/l). Ultrasound of the abdomen revealed massive splenomegaly (26.6 cm). Bone marrow examination showed a 80% infiltration of malignant lymphoid cells and impaired hematopoiesis. Flow cytometry, cytology, and histology confirmed the diagnosis of BRAF-V600E mutated classic hairy cell leukemia (HCL). HCL is identified by a unique cell morphology as well as the expression of specific surface markers (CD19, CD20, CD22, CD25, and CD11c) and most characteristic CD103 expression [1]. Moreover, the BRAF gene is mutated in 70–100% of HCL leading to consecutive RAF-MEK-ERK activation [1, 2]. Treatment with purine nucleoside analogs (PNAs), CD20 antibody therapy, and targeted BRAF inhibition are therapeutic options in the treatment of HCL, each with reasonable response rates but different myelotoxic potential [3]. Symptomatic anemia would have required the transfusion of red blood cells and the initiation of a cytostatic treatment regimen, but due to her religious beliefs as a member of the Jehovah’s Witnesses (JW), the patient denied blood transfusions. Most likely, due to the strong infiltration of the bone marrow with HCL cells, an initial attempt to raise RBC with erythropoietin failed. To spare hematopoietic function, a schedule containing PNAs was postponed, and rituximab treatment (375 mg/m^2^ weekly) was initiated leading to incomplete hematological response. Since PNA treatment could have caused fatal myelotoxicity and BRAF V600E mutation was confirmed, vemurafenib treatment was initiated (960 mg twice daily) and led to excellent hematologic response, but its use was limited by toxic side effects affecting the skin after two months of treatment. It was now feasible to administer cladribine in a dose-reduced schedule (80% dose) as definitive treatment, but G-CSF-refractory, reduced leukocytes, as well as ANC were suspicious for residual disease, which was confirmed by flow cytometry of the peripheral blood (0.15% of all events) as well as bone marrow examination (0.4% of all events by flow cytometry, 18% of nucleated cells by histology). Finally, a second course of cladribine, now in full dosage, led to complete response (Fig. 1).

**Fig. 1:**
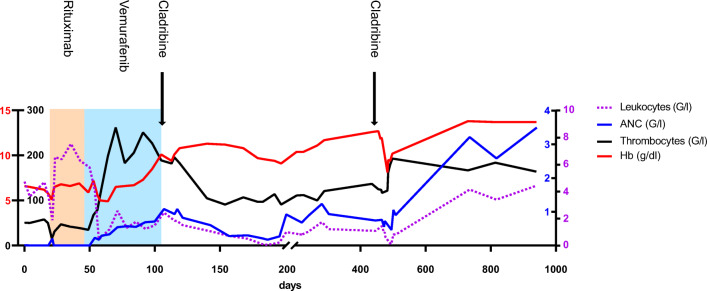
Time course of hemoglobin concentration (Hb, red), absolute neutrophil count (ANC, purple), absolute leukocyte count (blue), and thrombocyte count (black). Treatment with rituximab, vemurafenib, and cladribine is marked in the graph

In conclusion, we report a case of a JW patient with the diagnosis of HCL and compromised red blood cell and neutrophil counts due to extensive infiltration of the bone marrow. Since JW refuse the transfusion of allogeneic blood products and standard therapy containing PNAs carries the risk to cause fatal, life threatening anemia, a personalized treatment concept is needed for these patients. Although BRAF inhibition failed to induce a complete remission in our patient, we want to highlight its potential as an initial treatment of HCL in JW patients with significant bone marrow infiltration. Moreover, in this specific case, as well as in accordance with the data analyzed in the meta-analysis [3], BRAF inhibition seems to be more potent than a regime only containing rituximab. Hence, BRAF inhibitors might help to improve anemia and thrombocytopenia in JW patients and serve as a successful bridging concept until blood counts allow applying a myelotoxic cytostatic therapy.

## Data Availability

Data is available upon request.
